# Increasing the throughput of crystallization condition screens: Challenges and pitfalls of acoustic dispensing systems

**DOI:** 10.1016/j.mex.2019.09.030

**Published:** 2019-09-25

**Authors:** Robin Kryštůfek, Pavel Šácha

**Affiliations:** Institute of Organic Chemistry and Biochemistry, Academy of Science of the Czech Republic, Flemingovo n.2, 16610 Prague 6, Czech Republic

**Keywords:** Crystallization screening using acoustic dispensing, Acoustic liquid transfer, Protein crystallography, Crystallization screening, Laboratory automation

## Abstract

Advances in contactless acoustic liquid transfer technologies have unlocked opportunities to substantially increase the throughput of crystallization screens and decrease the consumption of reagents and consumables. Acoustic energy transfer enables crystallization experiments to be set up precisely and rapidly on a nanoliter scale. Nonetheless, adapting acoustic transfer methods to a diverse range of crystallization conditions and their physicochemical idiosyncrasies remains a major bottleneck for true universality of this technique. Even though the reagent limitations still remain an issue, we present a straightforward protocol for setting up crystallization experiments by acoustic transfer using a Labcyte Echo 550 instrument, with a focus on the technical limitations of this method, including reagent compatibilities, spatial resolution and downscaling limits.

• Set up crystallization screens in a small scale with reliable drop volumes as low as 50 nl
• Overview of commonly used crystallographic screen compatibility with acoustic dispensing
• Comparison of instrument calibrations and settings and its effects on error rate and screen reproducibility

## Graphical abstract

**Figure:**
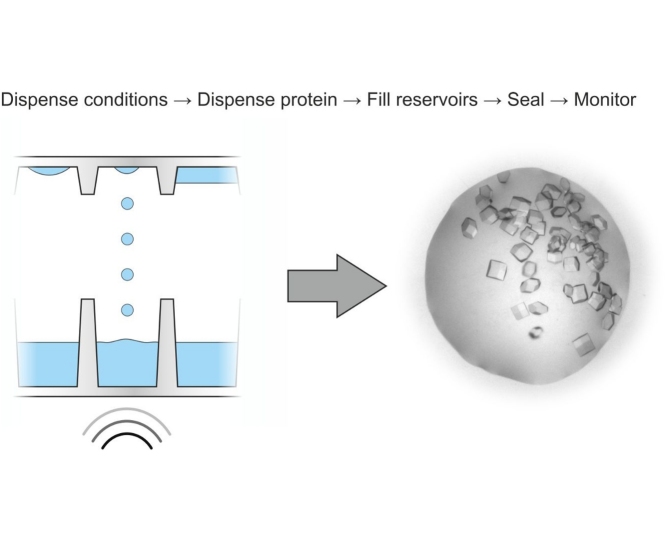

**Specification Table**

**Table:** 

Subject Area:	Biochemistry, Genetics and Molecular Biology
More specific subject area:	Protein crystallography
Method name:	Crystallization screening using acoustic dispensing
Name and reference of original method:	Acoustic liquid transfer Ellson, R., et al., Transfer of Low Nanoliter Volumes between Microplates Using Focused Acoustics—Automation Considerations. JALA: Journal of the Association for Laboratory Automation, 2003. 8(5): p. 29-34. Crystallization screening using acoustic dispensing Villasenor, A.G., et al., Nanolitre-scale crystallization using acoustic liquid-transfer technology. Acta Crystallographica Section D, 2012. 68(8): p. 893-900.
Resource availability:	Labcyte Echo 550 liquid handler: https://www.labcyte.com/products/liquid-handling/echo-550-liquid-handler Labcyte Echo Plate Reformat software: https://www.labcyte.com/products/software/echo-plate-reformat

## Method details

### Materials and hardware

• Acoustic dispensing system

In this article, we focus on the Labcyte Echo 550, but crystallization condition compatibility and some technical details are discussed for the Labcyte Echo 550 and EDC Biosystems ATS-100 systems as well.

• 384-well polypropylene Echo qualified source plate (Labcyte PP-0200)

The 384PP plate has a dead volume of 20 μl, which may be limiting if insufficient protein material is available. In that case, consider switching to 384-Well Low Dead Volume (LP-0200) or 1536-Well Low Dead Volume (LP-0400) plates, which have dead volumes of 6 and 1 μl, respectively.

• Sitting drop crystallization destination plate

We typically use Swissci 96-well 3-drop low profile plates (Hampton research HR3-205; geometry available in Supplementary Material). Special care must be taken to select plates with sufficiently low flange to prevent them from falling from the instrument locking mechanism.

• 96-well deep well block

This plate will be used for reservoir dispensing. We typically use the Nunc 96-Well Polypropylene DeepWell Block (Thermo Fisher 278752)

• Multichannel pipet and tips (10–100 μl)
• Crystallization screen conditions (see Table 1 for compatibility of commercial screens)

**Table 1:** Summary of the acoustic transfer compatibility of commercial crystallization screens. Locations of errors (i.e. conditions not transfered) in each screen are described in Fig. 1.

		Labcyte Echo 550		EDC Biosystems ATS-100		
Suite	Manufacturer	Errors	%	Errors	%	Reference
Crystal Screen HT	Hampton Research	0	100%	–	–	[4,6]
PEG/Ion HT	Hampton Research	0	100%	–	–	[4,6]
Index HT	Hampton Research	1	99%	8	92%	[4,5,6]
Additive Screen	Hampton Research	1	99%	–	–	[4]
Wizard I&II	Emerald Biosciences	0	100%	7	93%	[4,5,6]
ComPAS Suite	Qiagen	1	99%	–	–	[4,6]
PACT Suite	Qiagen	0	100%	–	–	[4]
JCSG+	Nextal	–	–	11	89%	[5]
Classics	Nextal	–	–	7	93%	[5]
JCSG++	Jena Bioscience	3	97%	–	–	This work
PACT++	Jena Bioscience	0	100%	–	–	This work
Morpheus	Molecular Dimensions	0	100%	–	–	This work
MIDASplus	Molecular Dimensions	19	80%	–	–	This work
• Protein sample (2.4 μl for a 96-condition screen at 50 nl scale)
• Plastic or glass sheet (130 × 85 mm or larger) for temporary coverage of destination plates
• Microplate sealing foil

Greiner EASYseal sealing film (Sigma A5596) is one option. Exercise caution when selecting proper sealing foil if UV-transparency is required. To secure the seal, we use 3 M PA-1 wallpaper soft scrapers.

### Preparative work

• Set up the destination plate geometry in the instrument control interface.

An example of the geometry settings for the Labcyte Echo 550 system with Swissci 96-well 3-drop low profile plates is available in Supplementary Material Table S1. This 3-drop plate allows use of the standard 384 layout for A1 and B1 stages with X −800 μm offset for stage B2. Some crystallization plates do not form a regular well matrix and thus require definition of additional manual offsets.

• Prepare the source plate with crystallization conditions and seal the plate if not using immediately.

A single 384 P P Echo plate can be used for four 96-well screens. For facile preparation and subsequent use, transfer each 96-well grid in a separate quadrant (A1,A2,B1,B2). Check the recommended screen storage conditions to verify whether they can be stored on a single plate. For commercial screen compatibility, see Table 1.

• Prepare a separate 96-well block with screen conditions for reservoir dispensing.
• Check the coupling fluid system thoroughly for air bubbles. Change coupling media, clean filters, and purge if necessary. Perform transducer focus calibration.

Transitional inconsistencies in transfer are chiefly caused by disruptions in the water coupling interface. Focus calibrations should be performed regularly to ensure the system is running properly.

• This protocol is designed for filling the reservoir well after dispensing the protein-condition droplets. It is possible to dispense the reservoir contents beforehand, while skipping step 8. In this case, a lower reservoir volume is necessary, as the Swissci 96-well 3-drop low profile plates reservoir can only hold up to 20 μl in the upside-down orientation. Always check if your screen conditions remain in the wells after turning the plate.

## Procedure

1 Equilibrate the prepared condition source plate to room temperature if necessary.
2 Dispense the protein sample into a single well in an Echo-compatible plate.

For information on plate selections and dead volumes, see Materials section.

3 Centrifuge the plates briefly (2 min, 1500 × g) to collect condensed fluid on the seal and level the well contents.

It is imperative to centrifuge the source plates before use to avoid transfer inconsistencies and intermittent errors.

4 Set up the screen condition transfer protocol.

For Echo 550 Plate Reformat, use a new Protocol with Custom Mapping mode combined with a Replication with Source and Destination interleave to transfer a single screen set quadrant to the desired plate sub-well (for graphical description of the workflow, see Supplementary Material Fig. S1). Successful acoustic transfer relies on proper selection of ultrasonic transducer power calibrations which are designed for specific sample compositions. Among the available instrument calibration settings, 384PP_AQ_CP (calibration for 384-well polypropylene plates and aqueous crystallization conditions) exhibited the lowest error rate with diverse sets of crystallization conditions (albeit at the cost of longer transfer time). For comparison, see Table 2.

**Table 2:** Effects of different Echo 550 instrument calibration settings on transfer time (net from instrument log) and error rate on a sample in-house PEG crystallographic screen (96 conditions, 5 nl per well; error number and transfer time were identical in triplicate measurements).

Calibration	Transfer time	Errors
384PP_DMSO2 (DMSO)	34 s	7 (7.3%)
384PP_AQ_SP2 (Aq.)	29 s	3 (3.1%)
384PP_AQ_GP2 (Glycerol)	26 s	21 (21.9%)
384PP_AQ_BP2 (Glycerol)	30 s	5 (5.2%)
384PP_AQ_CP (CP-Buffer)	59 s	0

It is advisable to use a final drop size of at least 50 nl (i.e., 25 nl condition volume for a 1:1 v/v ratio). Drops of volume less than 50 nl are prone to skin formation and premature drying during preparation. Once the drops are in a sealed system with vapor pressure maintained by the reservoir, they should be safe from drying (but not necessarily from skin formation, which is heavily dependent on the specific assayed protein) for as long as the plate sealing remains secure (up to a year), regardless of drop volume. For an example of the effects of varying drop size under the same conditions, see Fig. 2.

5 Set up the protein transfer protocol analogously to the previous step.

Protein samples can generally be safely transferred using faster calibration settings, such as 384PP_AQ_BP2.

6 Transfer screen conditions using the protocol set up in step 4.

Cover the plate immediately after it is ejected from the instrument using a sheet of glass or plastic to limit evaporation.

7 Add protein solution to condition drops using the protocol established in step 5.
8 Fill reservoir wells from the prepared 96-well storage block – 30 μl per reservoir.

While it is possible to dispense reservoir wells with Labcyte Echo 550, it is not advisable due to (i) the long transfer times for large volumes, (ii) the limited amount of fluid that the destination plate can reliably hold in an upside-down orientation (up to 20 μl in Swissci 96-well 3-drop low profile plates), and (iii) the fact that the 384PP source plate can hold enough volume for only 2 plates with 20 μl including dead volume.

9 Seal the plate using microplate sealing foil.

## Method validation

**Fig. 1:**
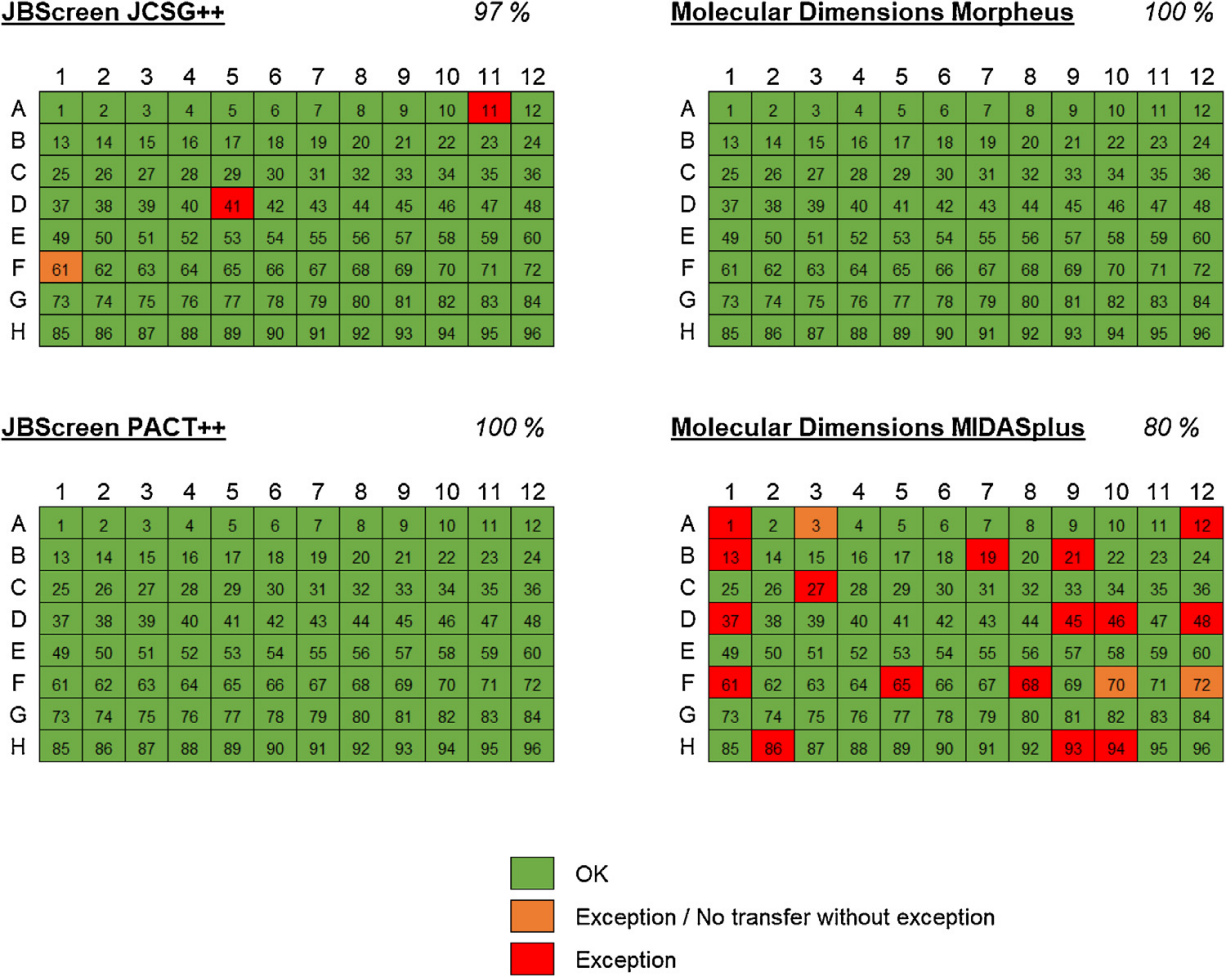
Acoustic transfer compatibility heatmap of several common crystallization screens. Green cells indicate successful transfer, red cells indicate no transfer with exception raised by the instrument, and yellow cells indicate no transfer, but only with intermittent instrument exception reporting. Compositions of all assayed conditions are available in Supplementary Material.

We assessed the following four commonly used commercial crystallographic screens for compatibility: JBScreen JCSG++ and JBScreen PACT++ from Jena Bioscience and Morpheus and MIDASplus from Molecular Dimensions (see Fig. 1). Other than MIDASplus, which had a 20% error rate, the screens exhibited an acceptable error rate less than 3%. Acoustic transfer of several classes of compounds was found to be problematic; these included high concentrations of MPD (consistent with the literature [5]), polypropylene glycol 400 (44% error rate) and its bis(2-aminopropyl ether) derivatives PPGBA (58% error rate), and multiple SOKALAN polymers (27% error rate, chiefly CP5, CP45, PA25CL and HP56). It is important to note that some transfer errors do not report an exception consistently, stressing the need for a thorough visual inspection during protocol optimization. However, this behavior was observed only in less than a fifth of total transfer errors.

**Fig. 2:**
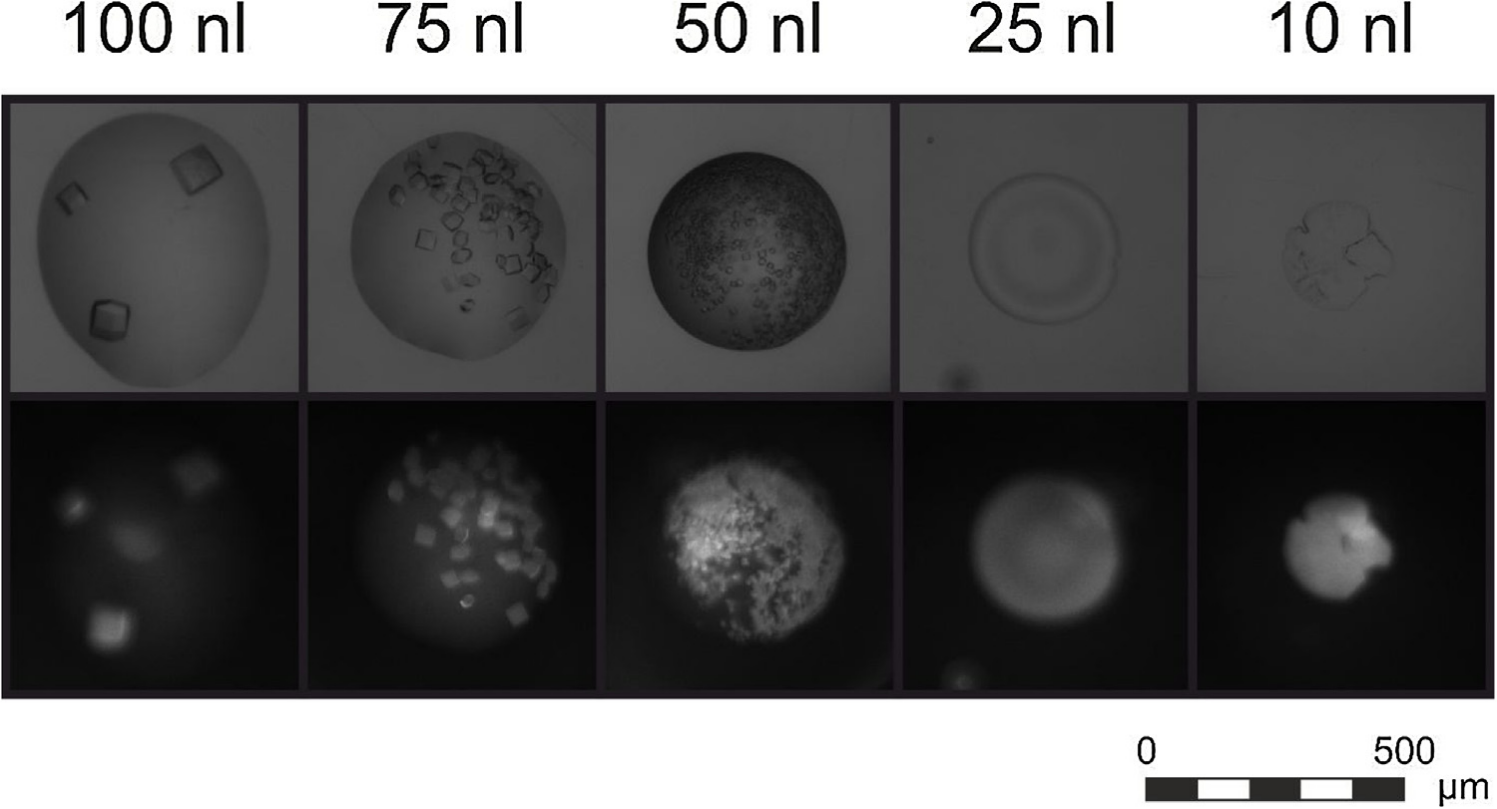
Effects of varying final drop volumes under equal conditions (1:1 v/v of 50 mg/ml chicken egg lysozyme with 15% v/v Jeffamine® ED-2003, 10% v/v ethanol). First row – visible light, second row – UV fluorescence.

**Fig. 3:**
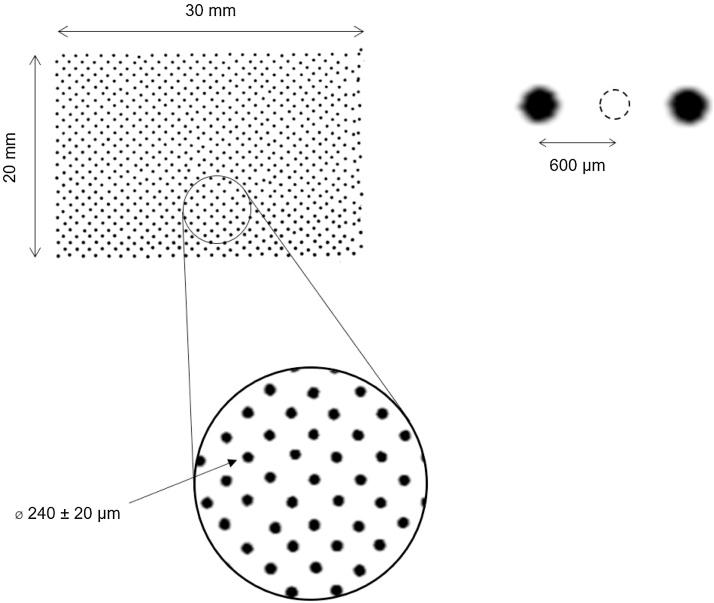
Illustration of spatial transfer accuracy using a checkered 32 × 48 matrix on a 20 × 30 mm nitrocellulose membrane (40 DPI). A single 2.5 nl drop of 1 mg/ml BSA-FITC was deposited on each spot, and the membrane was subsequently read with a fluorescence scanner.

When handling small drops, the spatial accuracy of the transfer is essential. Echo 550, in our hands, suffers from drop position errors (for illustration, see Fig. 3). However, these errors are consistent and mostly do not cause formation of multiple drops (see Supplementary Material Fig. S2). Depending on the toolpath generated from the protocol (the order in which the wells are transfer, and in turn how the plate move during the transfer protocol), spatial errors chiefly accumulate on the edges of the dispensed array, likely due to vibrations caused by simultaneous movements of both the x and y instrument axes. These errors can be mitigated to a degree by introducing a dispensing delay after each well in the protocol setup.

Another issue when setting up screens is evaporation. While conventional crystallographic screening instruments (e.g., Douglas Instruments Oryx8) use sliding evaporation shields to alleviate this issue, Echo 550 inadvertently prevents it by increased humidity in the instrument due to constantly exposed coupling liquid. While drop evaporation can be mitigated by working with sufficiently high drop volumes and covering the plate between preparation steps, attention should be paid to evaporation of conditions from source plate. Resulting increase of viscosity over time can lead to emergence of more transfer errors.

### Additional information

Since the acoustic transfer method was first introduced as a viable option for contactless liquid transfer in laboratory settings [1], there have been multiple attempts to comprehensively describe its technical limits for use in crystallization screening [[2], [3], [4], [5]]. In the meantime, dedicated instruments using this technology have become available from two major companies (Labcyte and EDC Biosystems), and thus the technique has ceased to be available only to niche users and become a generally available option for liquid handling. Implementation of acoustic transfer using the Labcyte Echo 550 instrument has significantly increased the throughput of our crystallization trials and allowed us to streamline the screening preparation process.

As with any advanced instrumental method, it is imperative to exercise proper instrument care and regular maintenance to avoid transfer inconsistencies. Caution must be taken when using screens containing large concentrations of MPD [5], several polymer additives discussed above. Nonetheless, acoustic transfer using Labcyte Echo 550 enables swift setup of crystallization experiments and offers the major advantages of contactless transfer and thus a low probability of cross-contamination, small scale with reliable drop volumes as low as 50 nl, and a high degree of reproducibility.
