# Supplementary material for: Increasing the throughput of crystallization condition screens: Challenges and pitfalls of acoustic dispensing systems
Source: MethodsX. 2019 Sep 25;6:2230–6. doi: 10.1016/j.mex.2019.09.030 (PMC6812406; doi:10.1016/j.mex.2019.09.030)
Supplement: Supplementary file 1 [file mmc1.docx]

**Supplementary Material**

**Increasing the throughput of crystallization condition screens: Challenges and pitfalls of acoustic dispensing systems**

*Robin Kryštůfek, Pavel Šácha*

Institute of Organic Chemistry and Biochemistry, Academy of Science of the Czech Republic, Flemingovo n.2, 16610 Prague 6 (Czech Republic)

**
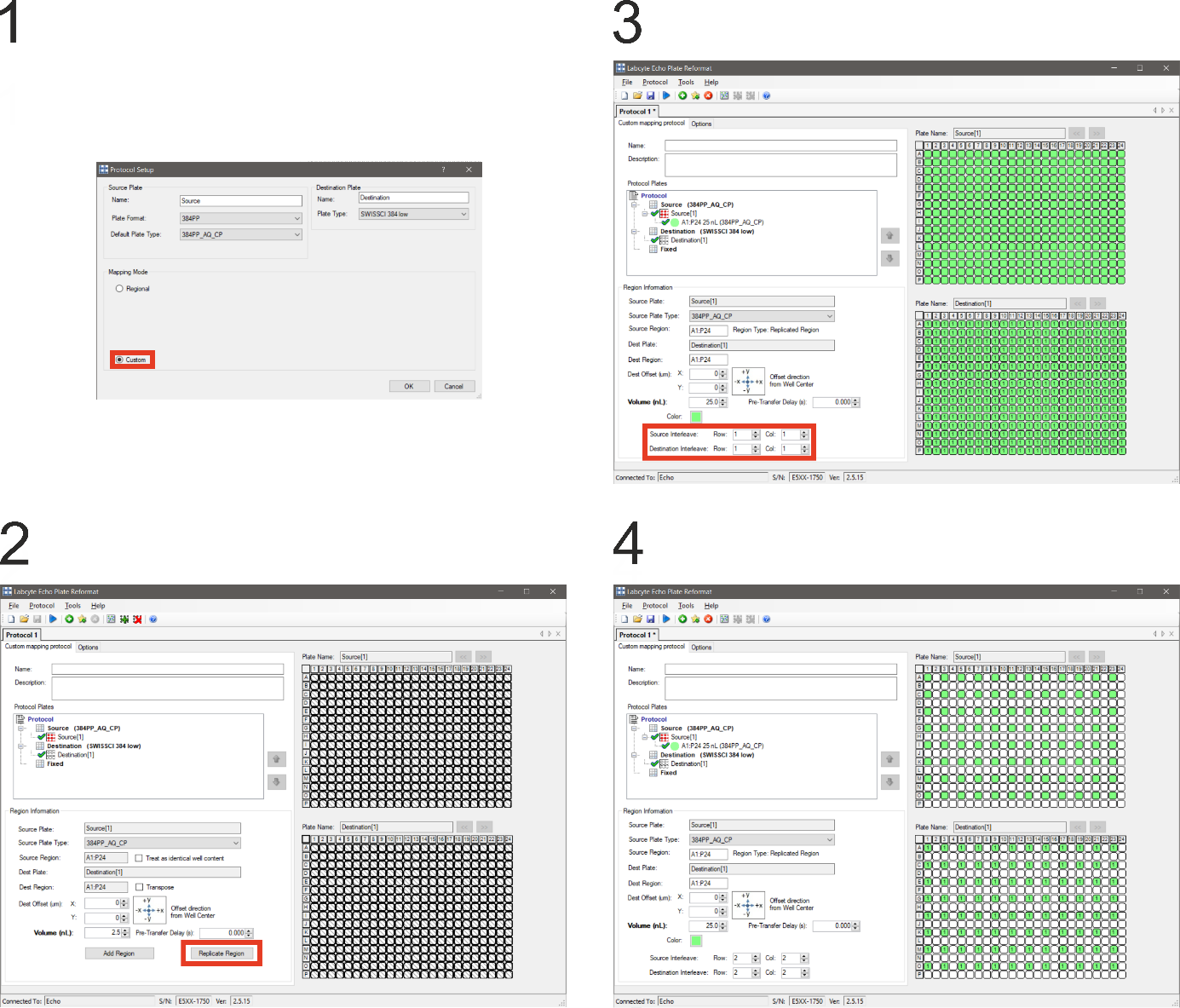
**

**Figure S1.** Setup of condition transfer protocol in Echo 550 Plate Reformat. (1) Create a new Protocol with Custom Mapping mode. (2) Select the entire plate area of both source and destination plate and designate a Replication region transfer. If you need to use different source or destination quadrant, shift the selection so that it begins in the first quadrant well. (3) Set Source and Destination interleave to two in order to transfer a single screen set quadrant to the desired plate sub-well. (4) Resulting transfer pattern.


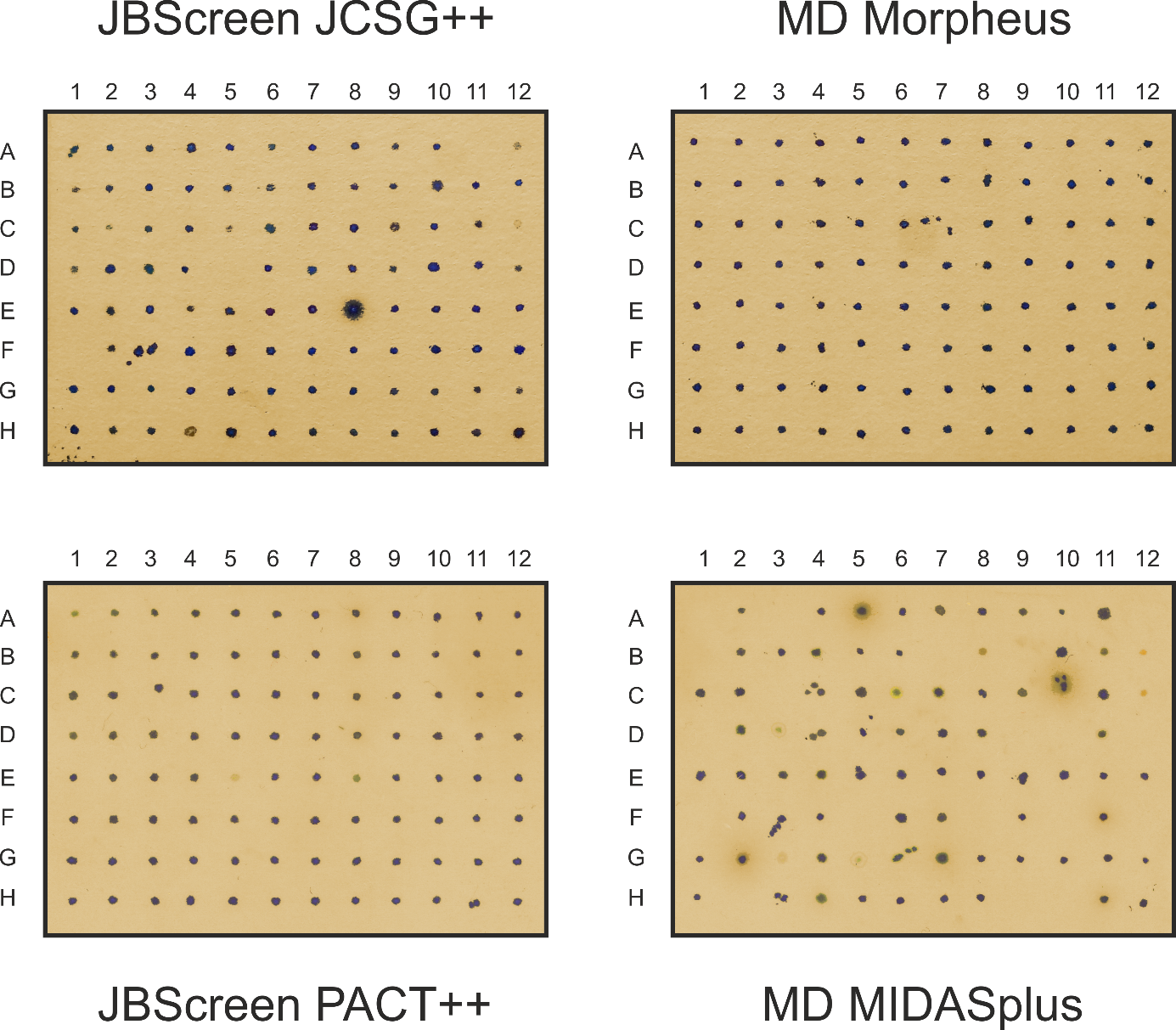


**Figure S2.** Transfers of 15 nl (8 drops) of crystallization matrix conditions to water-sensitive paper.

| Parameter | Value |
| --- | --- |
| Number of Rows | 16 |
| Number of Columns | 24 |
| A1 X Offset (A) | 12.4 mm |
| A1 Y Offset (B) | 9.20 mm |
| X Center Spacing (C) | 4.51 mm |
| Y Center Spacing (D) | 4.49 mm |
| Plate Height (E) | 7.80 mm |
| Flange Height (F) | 2.50 mm |
| Well Width (G) | 3.00 mm |
| Well Length (G) | 3.00 mm |
| Well Capacity (H) | 1.00 μl |

**Table S1.** Swissci 96-well 3-drop low profile plate (Hampton research HR3-205) geometry on Labcyte Echo 550. Stages in B2 quadrant require a manual X offset of -800 μm (see Figure S1 1-3 on the bottom left).
